# Influence of miR-101 on proliferation of liver cancer cells through the MAPK/ERK signaling pathway

**DOI:** 10.3892/ol.2019.11210

**Published:** 2019-12-12

**Authors:** Xuan Meng, Yong Shi, Xin Xiang, Chonghui Li, Xinlan Ge, Ke Pan, Yurong Liang

**Affiliations:** Department of Hepatobiliary Surgery, Chinese PLA General Hospital, Beijing 100853, P.R. China

**Keywords:** miR-101, liver cancer, EZH2, ERK, proliferation

## Abstract

The expression of miR-101 in carcinoma and para-carcinoma tissues of patients with liver cancer was studied. The carcinoma and para-carcinoma tissues of 67 patients with liver cancer treated in Chinese PLA General Hospital were collected, and the expression of miR-101 in carcinoma and para-carcinoma tissues was detected via reverse transcription-polymerase chain reaction (RT-PCR). The liver cancer HepG2 cell line was transfected with miR-101 mimics. Moreover, the influence of miR-101 overexpression on the proliferation of liver cancer cells was detected via Cell Counting Kit-8 assay and colony formation assay. The proportion of Ki67-positive cells in the control group (NC group) and miR-101 overexpression group (miR-101 mimics group) was detected via Ki67 staining. The proportions of cells were detected via flow cytometry, and the predicted target gene Zeste2 enhancer (EZH2) was further verified via luciferase reporter gene assay and western blotting. The miR-101 overexpression significantly inhibited the colony formation and proliferation ability of liver cancer cells (P<0.05). The proportion of Ki67-positive cells in liver cancer cells was lower in miR-101 mimics group (P<0.05). The proportion of cells in G0/G1 phase was increased in miR-101 mimics group compared with that in NC group (P<0.05). The extracellular signal-regulated kinase (ERK)1/2 phosphorylation level in liver cancer cells was obviously suppressed in miR-101 mimics group (P<0.05). Therefore, the expression level of miR-101 declines in liver cancer tissues, and the miR-101 overexpression can inhibit the proliferation of liver cancer cells. The inhibitory effect of miR-101 on the proliferation of liver cancer cells may be related to its inhibition on the mitogen-activated protein kinase (MAPK)/ERK signaling pathway, and the inhibition on the MAPK/ERK may be mediated by the targeted inhibition of miR-101 on EZH2.

## Introduction

Primary liver cancer is one of the six most common cancers, and the number of deaths ranks 2^nd^ in the total cancer-related deaths (1). Hepatocellular carcinoma, the most important primary liver cancer, accounts for approximately 90% in primary liver tumors, making it a major international public health issue (2). The occurrence and development of liver cancer is a progressive cumulative complex process covering multiple factors, stages, mechanisms, links and genetic changes, which involves a variety of abnormal cellular or molecular changes, such as oxidative stress, endoplasmic reticulum stress and cell cycle disorder (3,4). Therefore, clarifying the molecular mechanism for the occurrence and development of liver cancer has great significance in its early diagnosis and treatment.

The polycomb group (PcG) protein is an important epigenetic regulatory factor, which can serve as a transcription inhibitor silencing specific genomes via chromatin modification (5). The PcG protein belongs to the polycomb repressive complex (PRC) family. PRC2 includes the Zeste2 enhancer (EZH2), suppressor of Zeste12 (SUZ12) and embryonic ectoderm development (EED) (6). EZH2 is a catalytically active component of PRC2, which can catalyze the histone H3 lysine 27 (H3K27) for trimethylation after forming the complex with EED (7). Recently, increasingly more studies have revealed that EZH2 has a cancer-promoting effect, including the induction of the abnormal cell differentiation and promotion of cancer cell proliferation (8). EZH2 is overexpressed in various tumors, showing a close correlation with the poor prognosis of patients (9). It is reported in studies that the low expression of miR-101 in glioma cells can lead to the upregulation of EZH2, thereby enhancing the proliferation, invasion and migration of glioma cells (10).

miRNAs are a group of single-stranded non-coding RNAs existing in eukaryotes, with 20–24 nt in length and various regulatory functions (11). miRNAs can regulate the expression of a variety of genes through targeted binding to specific genes, thus playing an important role in the physiological activities of cells, such as proliferation, differentiation and apoptosis (12). To the best of our knowledge, the expression of miR-101 in liver cancer and its mechanism have not been reported yet. In the present study, the expression level of miR-101 in carcinoma and para-carcinoma tissues of liver cancer patients was detected, and the liver cancer cell lines with miR-101 overexpression were constructed using miR-101 mimics, so as to observe the influence of miR-101 overexpression on the proliferation of liver cancer cells and further explore the potential mechanism of miR-101 in affecting the proliferation of liver cancer cells.

## Materials and methods

### 

#### Tissue specimens

A total of 67 pairs of liver cancer tissues and the corresponding para-carcinoma tissues surgically resected in Chinese PLA General Hospital (Beijing, China) from December 2016 to June 2018 were collected. After the blood stains were washed away with normal saline, all specimens were cut into pieces, placed into an Eppendorf (EP) tube and stored in a refrigerator at −80°C. All the above procedures were approved by the Medical Ethics Committee of Chinese PLA General Hospital and informed consents were signed by the patients or the guardians.

#### Cell culture

The liver cancer HepG2 cell line was purchased from the Biological Research Institute of the Chinese Academy of Sciences (cat. no. TCHu106). Phosphate buffered saline (PBS), trypsin, fetal bovine serum (FBS) and RPMI-1640 medium were purchased from Gibco; Thermo Fisher Scientific, Inc. Small interfering RNAs (siRNAs) were from Google Biology. HepG2 cells were cultured in an incubator with 5% CO_2_ at 37°C, and then digested with 0.25% trypsin-EDTA and passaged when they fully covered the culture dish.

#### Detection of expression of related genes via reverse transcription-polymerase chain reaction (RT-PCR)

i) The total RNA was extracted from liver cancer and para-carcinoma tissues using TRIzol (RT kit cat. no. 10928042), the concentration and purity of the RNA extracted were detected using an ultraviolet spectrophotometer (Mettler Toledo) and the RNA with absorbance (A)260/A280 of 1.8–2.0 was used. ii) The messenger RNA (mRNA) was synthesized into the complementary deoxyribonucleic acid (cDNA) through RT kit (cat no. 4366596; Thermo Fisher), and stored in the refrigerator at −80°C. iii) RT-PCR system: 2.5 µl 10X buffer, 2 µl cDNAs, 0.25 µl forward primers (20 µmol/l), 0.25 µl reverse primers (20 µmol/l), 0.5 µl dNTPs (10 mmol/l), 0.5 µl Taq polymerase enzymes (2×10^6^ U/l) and 19 µl ddH_2_O. The amplification system of RT-PCR was the same as above. It was synthesized at 50°C and amplified (40 cycles). The final Cq value was measured by LightCycler 480 system. The internal reference was *GAPDH*. Primer sequence are shown in Table I.

#### Construction of cell lines with miR-101 overexpression

When HepG2 cells were in the logarithmic growth phase, they were immediately digested and inoculated into a 6-well plate. After 12 h (60–80% cells were fused), the complete medium was discarded, and cells were washed with the serum-free medium 2–3 times and starved in the incubator for synchronous growth. miR-101 mimics were dissolved in RNase deionized water to be prepared into transfection solution at a final concentration of 20 µmol/l. The cells were divided into the blank control group (NC group) and miR-101 overexpression group (miR-101 mimics group). The transfection solution prepared already was added into each well and fully mixed, followed by cell culture for another 6 h. Then the solution was replaced with complete medium.

#### Western blotting

The medium was discarded and washed by PBS three times. Each dish was filled with 1,000 µl RIPA lysis buffer (Thermo Fisher) and shaken for 20 min. Then the cells on the bottom of the dish were fully scraped off by a brush and put into the EP tube. The collected cells were lysed for about 15 sec with an ultrasound lyser and centrifuged for 0.5 h (at 10,000 × g) after 15 min at 4°C. The supernatant was separated into EP tubes, and the protein concentration was measured by BCA and ultraviolet spectrophotometry. The protein concentration of all samples was fixed to the same concentration. After packing, they were put in −80°C refrigerator. Then, 15% SDS-PAGE electrophoresis was performed after the total protein was extracted from liver cancer cells prior to the addition of 10 μg protein per lane. The protein was transferred to PVDF membrane. The membrane was blocked with 5% milk at 23°C for 1 h. Enhanced chemiluminescent (ECL) kit (Beyotime) was used for visualisation. Western blot analysis was carried out. An Odyssey scanner (Odyssey) was used to scan and quantify protein bands, and GAPDH was used to correct the protein level. Image J (NIH) was used for densitometry of the bands.

#### Cell Counting Kit-8 (CCK-8) cell proliferation assay

The cells in the logarithmic growth phase in each group were inoculated into a 96-well plate and cultured in the incubator with 5% CO_2_ at 37°C for 0, 24, 48, 72 and 96 h. The blank control group was used as the negative control group (NC group). Then the medium was discarded, and the developing solution was prepared in a dark place using RPMI-1640 medium and CCK-8 (10:1). Then, 110 µl developing solution was added into each well of the 96-well plate, followed by incubation at 37°C for 2 h, and the absorbance in each group was detected at 540 nm using the ultraviolet spectrophotometer. The experiment was repeated three times.

#### Ki67 staining

At 48 h after transfection with miR-101 mimics, liver cancer cell lines were stained using the Ki67 staining kit (Invitrogen; Thermo Fisher Scientific, Inc.) according to manufacturers instructions. After staining, the cells were photographed under a fluorescence microscope (Olympus) and three fields of view were randomly selected in each glass slide. Finally, the Ki67-positive cells were counted and quantified.

#### Detection of cell cycle via flow cytometry

The cells in the logarithmic growth phase were taken, digested with 0.25% trypsin-EDTA, prepared into suspension and inoculated into the 6-well medium. The cells were loaded and the proportions of cells in different phases were detected according to the instructions of the Annexin V-FITC PI cell cycle assay kit (Beyotime Institute of Biotechnology).

#### Luciferase reporter gene assay

First, the possible binding sites of transcription factors in the promoter region were predicted using bioinformatics method (TargetScan). The primers were designed, and the EZH2 gene segment was cloned from genomic DNAs via PCR and inserted into the luciferase reporter gene plasmid. The positive clones were screened. The miR-101 plasmid was amplified and purified for later use. At the same time, the corresponding empty plasmid control was set up. The reporter gene plasmid and transcription factor-expressing plasmid were co-transfected into cells. The specific fluorescein substrate enzyme was added, and the fluorescence intensity was detected to determine whether there was a targeted effect.

#### Statistical analysis

SPSS22.0 software (IBM Corp.) was used for the analysis of all data. Measurement data were expressed as mean ± standard deviation, and t-test was used for the comparison of data between the two groups. P<0.05 was considered to indicate a statistically significant difference.

## Results

### 

#### Expression of miR-101 in liver cancer and para-carcinoma tissues

**Figure 1.**
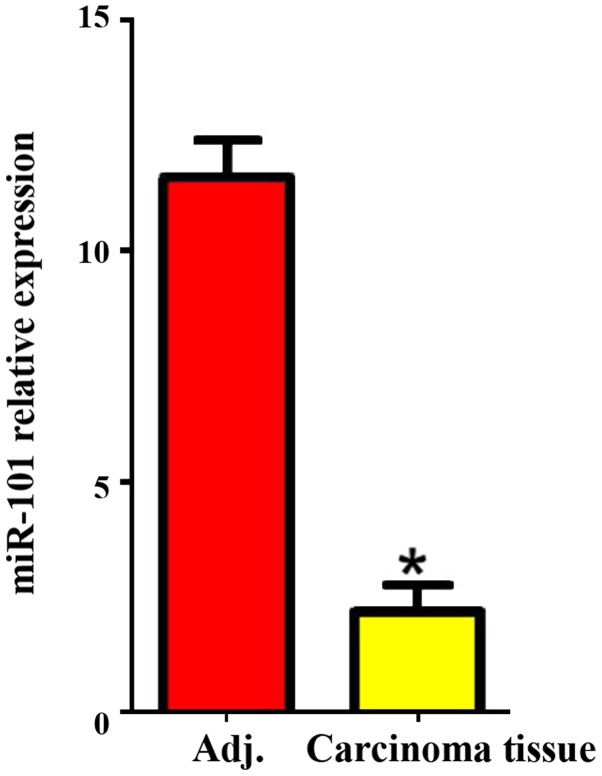
Expression of miR-101 in liver carcinoma and para-carcinoma tissues. Adj., para-carcinoma tissues. *P<0.05 (a statistically significant difference) vs. Adj.

The expression of miR-101 in carcinoma and para-carcinoma tissues was detected via RT-PCR, and the results revealed that the expression level of miR-101 in liver cancer tissues was significantly lower than that in para-carcinoma tissues (P<0.05; Fig. 1).

#### Influence of miR-101 overexpression on proliferation of liver cancer cells

**Figure 2.**
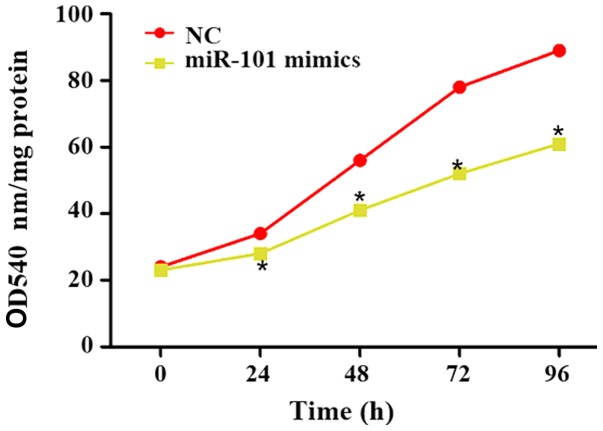
Influence of miR-101 overexpression on the proliferation of liver cancer cells. NC, blank control; miR-101 mimics, miR-101 overexpression. *P<0.05 (a statistically significant difference) vs. NC group.

At 0, 24, 48, 72 and 96 h after miR-101 mimics were transfected into liver cancer cells, the cell proliferation in each group was detected using the CCK-8 kit, and the optical density (OD) at 540 nm was used to reflect the proliferation ability. The results showed that the proliferation of liver cancer cells was significantly reduced at 0, 24, 48, 72 and 96 h after transfection of miR-101 mimics (P<0.05; Fig. 2).

#### miR-101 overexpression inhibits colony formation of liver cancer cells

**Figure 3.**
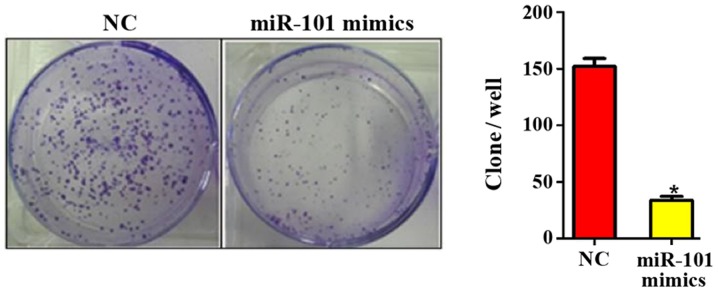
Influence of miR-101 overexpression on colony formation of liver cancer cells. NC, blank control; miR-101 mimics, miR-101 overexpression. *P<0.05 (a statistically significant difference) vs. NC group.

At 10 days after miR-101 overexpression, the colony formation ability in each group was detected. It was found that the number of colonies formed was 150.45±3.88 in NC group and 32.12±2.08 in miR-101 mimics group (P<0.05; Fig. 3), suggesting that the miR-101 overexpression can significantly inhibit the colony formation ability of liver cancer cells.

#### Ki67 staining results of miR-101 overexpression on liver cancer cells

**Figure 4.**
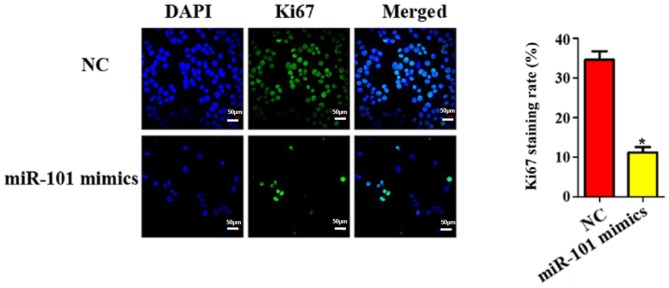
Influence of miR-101 overexpression on Ki67 staining of liver cancer cells. NC, blank control; miR-101 mimics, miR-101 overexpression. *P<0.05 (a statistically significant difference) vs. NC group.

Furthermore, the cell proliferation ability in each group was evaluated using Ki67 staining. As shown in Fig. 4, the transfection of miR-101 mimics was able to reduce the number of Ki67-positive cells by approximately 5.32 times (P<0.05).

#### Influence of miR-101 mimics on liver cancer cell cycle

**Figure 5.**
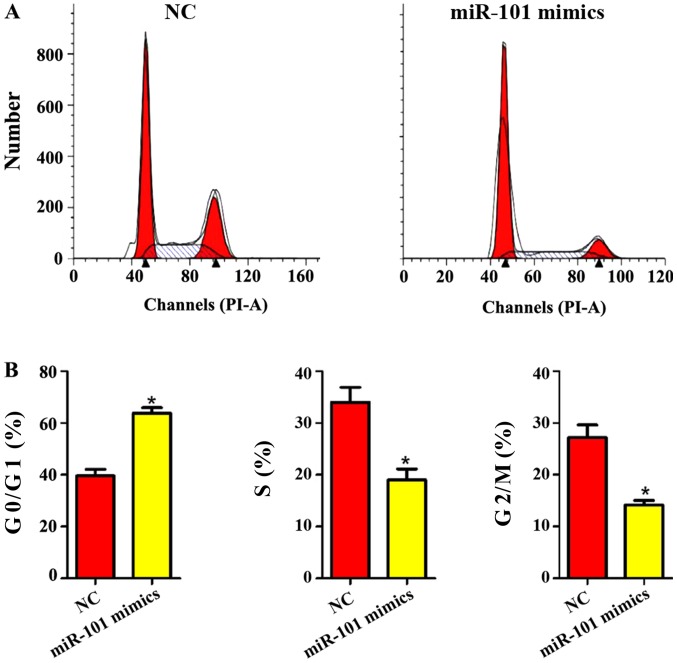
Influence of miR-101 overexpression on liver cancer cell cycle. (A) flow cytometry of cell cycle in the control group and miR-101 overexpression group; (B) the proportion of liver cancer cells in different phases. NC, blank control; miR-101 mimics, miR-101 overexpression. *P<0.05 (a statistically significant difference) vs. NC group.

As shown in Fig. 5, the liver cancer cell cycle was obviously changed after miR-101 mimics were added. In miR-101 mimics group, the proportion of liver cancer cells in G0/G1 phase was obviously increased, while the proportion of cells in G2/M and S phases was obviously decreased (P<0.05), indicating that miR-101 mimics can significantly inhibit the cycle of liver cancer cells.

#### Prediction and verification of miR-101 target genes

**Figure 6.**
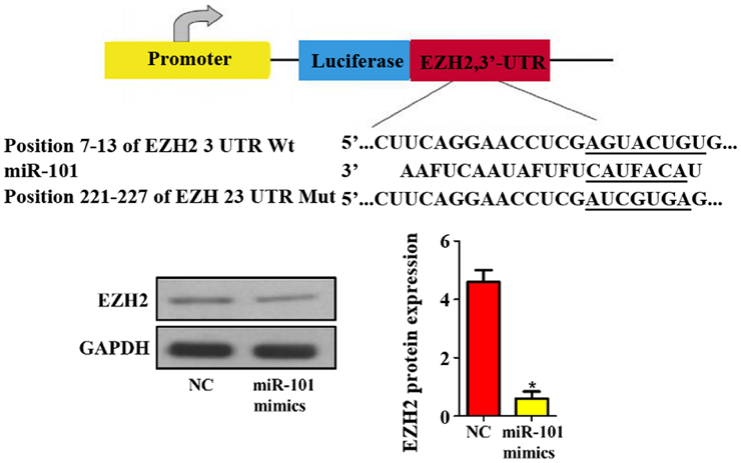
Prediction and verification of miR-101 target genes. NC, blank control; miR-101 mimics, miR-101 overexpression. *P<0.05 (a statistically significant difference) vs. NC group.

In addition, the target genes of mouse miR-101 were predicted using bioinformatics technique. The results revealed that EZH2 was one of the target genes of miR-101 (Fig. 6). Then the protein expression level of EZH2 in NC and miR-101 mimic groups was detected via western blotting, and it was found that miR-101 mimics remarkably suppressed the expression level of EZH2 in liver cancer cells compared with that in NC group (P<0.05; Fig. 6).

#### Influence of miR-101 overexpression on the mitogen-activated protein kinase (MAPK)/extracellular signal-regulated kinase (ERK) signaling pathway

**Figure 7.**
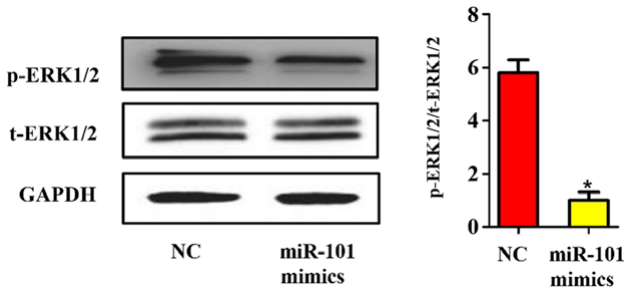
Influence of miR-101 overexpression on the MAPK/ERK signaling pathway. NC, blank control; miR-101 mimics, miR-101 overexpression. *P<0.05 (a statistically significant difference) vs. NC group.

Considering the important role of the MAPK/ERK signaling pathway in the occurrence and development of liver cancer, whether the activation of the MAPK/ERK signaling pathway in liver cancer can be regulated by miR-101 was detected. The ERK1/2 phosphorylated protein and total protein in each group were quantified using western blotting. As shown in Fig. 7, the phosphorylation of ERK1/2 was also significantly inhibited after miR-101 overexpression in liver cancer cells (P<0.05), further revealing that the inhibitory effect of miR-101 on the proliferation of liver cancer cells is realized by its inhibition on the MAPK/ERK signaling pathway through targeted binding to EZH2.

## Discussion

In recent years, the morbidity and mortality rates of liver cancer have increased year by year around the world, and the number of deaths is up to 662,000 every year, about half of which are from China (13). The main causes of liver cancer include hepatitis B virus infection, smoking and drinking. Therefore, many research efforts have been made to search for the pathogenic genes and diagnostic markers of liver cancer, such as the tumor size, alpha fetoprotein level and various differentially-expressed genes in primary liver cancer tissues (14). Despite the significant improvement in the diagnosis and treatment strategies, the overall prognosis of liver cancer is still poor (15). Therefore, it is of great significance to search for the key genes, proteins or RNAs causing liver cancer for the precise treatment of liver cancer.

With the rapid development of transcriptomics, increasingly more disease-related differentially-expressed genes have been revealed. Similarly, many studies have confirmed the role of miRNAs in the occurrence and development of liver cancer (16). For example, miR-139 can inhibit the invasion and metastasis of liver cancer cells through downregulating the expression of Rho-kinase 2 (17). On the contrary, miR-21 can promote the proliferation of liver cancer, whose mechanism may be related to the direct targeted inhibition of miR-21 on MAP kinase-kinase 3 (MAP2K3) (18). Moreover, miR-346 also serves as a cancer-promoting gene, which can facilitate the proliferation, invasion and metastasis of liver cancer cells through targeted inhibition on F-Box and leucine-rich repeat protein (FBXL2) (19). In the present study, it was found for the first time, to the best of our knowledge, that the miR-101 expression level in liver cancer tissues was significantly lower than that in para-carcinoma tissues, indicating that miR-101 may play a role as a cancer suppressor gene. In addition, the influence of miR-101 overexpression on the proliferation of liver cancer cells was detected using various molecular biological methods. It was proved in Ki67 staining, flow cytometry and colony formation assay that the miR-101 overexpression inhibited the cycle, DNA replication and division of liver cancer cells. EZH2 is a human gene discovered in recent years, which has a close correlation with the cell life activity. EZH2 can promote the proliferation and spread of tumor cells through inhibiting the characteristic target genes in chromatin, and the mechanism of its transcriptional inhibition may be related to its regulatory effect on histone methyltransferase (20). Cardenas *et al* found that inhibiting EZH2 can also promote the endothelial-mesenchymal transition of ovarian cancer, thereby inhibiting the invasion of ovarian cancer cells (21). It has been reported in previous studies that miR-101/EZH2 is expressed abnormally in a variety of tumors, including prostate cancer, bladder cancer, gastric cancer and glioma. Moreover, the abnormal expression of miR-101/EZH2 is closely related to migration, invasion and metastasis of these tumors (22–24). In the present study, it was primarily revealed, using bioinformatics and molecular biological methods, that EZH2 was one of the potential direct targets of miR-101. In fact, EZH2 can serve as a target gene for various miRNAs. For example, miR-98 can downregulate the Wnt/β-catenin signaling pathway through targeted inhibition on EZH2, ultimately suppressing the proliferation of liver cancer cells (25).

The MAPK/ERK signaling pathway plays an indispensable role in the biological behavior of liver cancer cells. It is reported that EZH2 can affect the activation of the MAPK/ERK signaling pathway, and the MAPK/ERK can also in turn affect the EZH2 expression, indicating that there may be a potential negative feedback regulatory correlation between EZH2 and MAPK/ERK. This study revealed that miR-101 also inhibited the phosphorylation activation of ERK1/2, but whether the activation of ERK1/2 depends on the expression of EZH2 remains to be further investigated. However, there are still some limitations in this study: i) only one kind of cell line was used; and ii) the subcutaneous tumor formation assay was not performed.

In conclusion, this study indicates for the first time to the best of our knowledge, that miR-101 can inhibit the phosphorylation level of ERK1/2 through targeted inhibition on EZH2, ultimately suppressing the proliferation of liver cancer cells.

## Figures and Tables

**Table I.** Primer sequences.

Target gene	Primer sequence
*GAPDH*	
Forward	5′-GACATGCCGCCTGGAGAAAC-3′
Reverse	5′-AGCCCAGGATGCCCTTTAGT-3′
*miR-101*	
Forward	5′-AAAGCTGATCGTAGGCTGTTCCTT-3′
Reverse	5′-AGTCGATGCCAAAGAAGT-3′

## Data Availability

The datasets used and/or analyzed during the current study are available from the corresponding author on reasonable request.
